# Supplementary material for: Manganese Dioxide Nanoparticles Prepared by Laser Ablation as Materials with Interesting Electronic, Electrochemical, and Disinfecting Properties in Both Colloidal Suspensions and Deposited on Fluorine-Doped Tin Oxide
Source: Nanomaterials (Basel). 2022 Nov 18;12(22):4061. doi: 10.3390/nano12224061 (PMC9698065; doi:10.3390/nano12224061)
Supplement: Supplementary file 1 [file nanomaterials-12-04061-s001.zip › nanomaterials-1928601-supplementary.pdf]

Supporting Information

Manganese dioxide nanoparticles prepared by laser ablation as materials with interesting electronic, electrochemical, and disinfecting properties in both colloidal suspensions and deposited on fluorine-doped tin oxide

Jhonatan Corrales[1], Jorge Acosta-Vergara[2], Sandra Castro[3], Henry Riascos[4], Efraím Serna- Galvis[5], Ricardo Torres- Palma[5], Yenny Ávila- Torres*[5]

[1] Maestría en Ciencias Químicas, Facultad de Tecnologías, Universidad Tecnológica de Pereira, Pereira, Colombia.

[2] Department of Macromolecular Compounds, Faculty of Chemistry, Lomonosov Moscow State University MSU, Russia, 119991, Moscow 1, GSP-1, 1-3 Leninskiye Gory

[3] Universidad Santiago de Cali, Pampalinda, Santiago de Cali, 760035, Colombia.

[4] Grupo de ablación láser, Universidad Tecnológica de Pereira, Barrio la Julita. Pereira, Colombia.

[5] Grupo de Investigación en Remediación Ambiental y Biocatálisis (GIRAB), Instituto de Química, Facultad de Ciencias Exactas y Naturales, Universidad de Antioquia UdeA, Calle 70 No. 52-21, Medellín, Colombia; Correspondence: yenny.avila@udea.edu.co, ricardo.torres@udea.edu.co

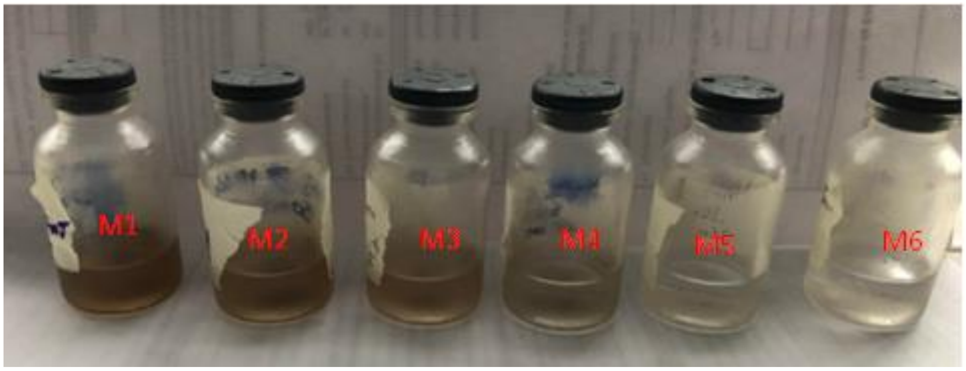

Figure S1. MnO_2 Nps in Methanol; in where are describe as:
M1: 80mJ, 10min; M2: 80mJ, 5 min; M3: 50mJ, 10 min; M4: 50mJ, 5 min; M5; 25mJ, 10 min; M6: 25mJ, 5 min).

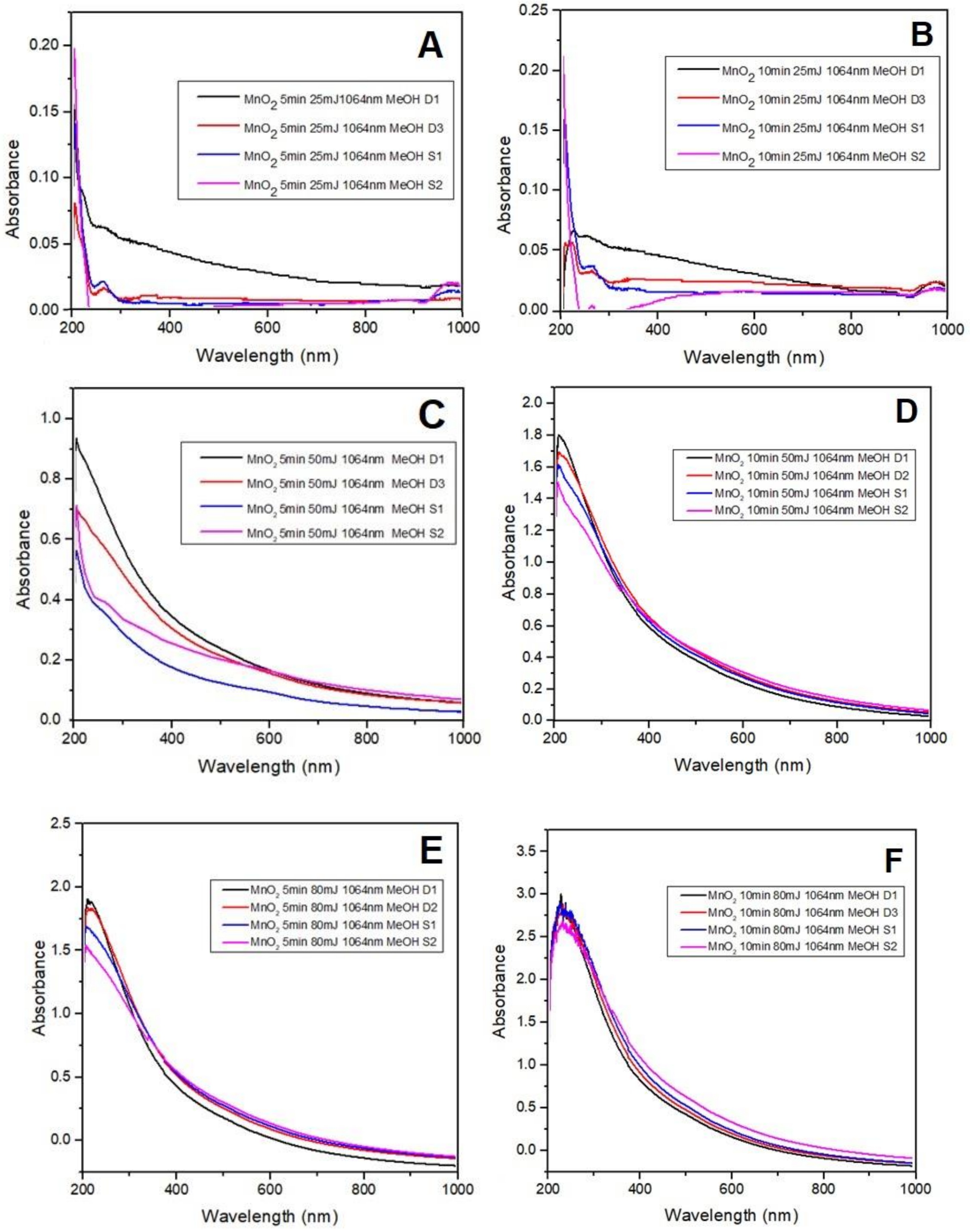

Figure S2. Electronic properties for MnO_2 NPs at different times. Laser ablation (1064 nm).

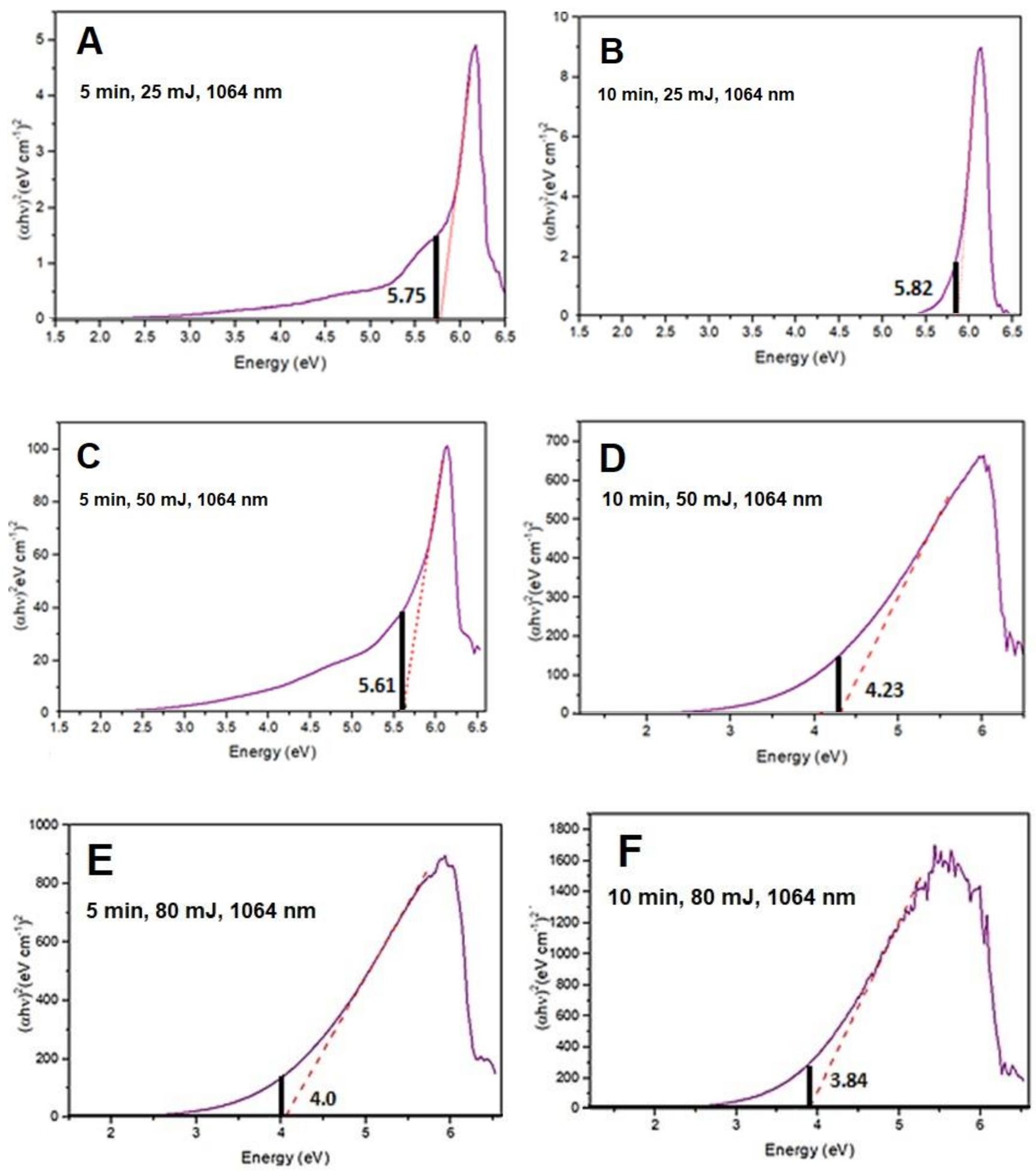

Figure S3. GAP determination for NPs MnO_2 at different times and energies.

Sample MnO_2 8/11/2022 4:01:17 PM

Spectrum processing:
Peaks possibly omitted: 1.488, 2.145, 3.698, 9.694 keV

Processing option: All elements analyzed (Normalized)
Number of iterations = 2

Standard:
O SiO2 1-Jun-1999 12:00 AM
Mn Mn 1-Jun-1999 12:00 AM

Element	Weight%	Atomic%
O K	79.32	52.77
Mn K	20.68	47.23
Totals	100.00	

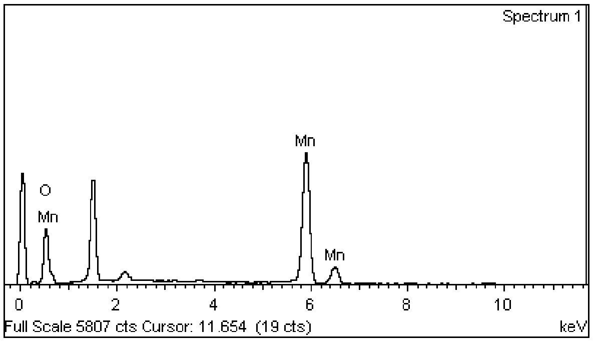

Figure S4. Element distribution map (EDX) of MnO_2 for SEM micrograph.

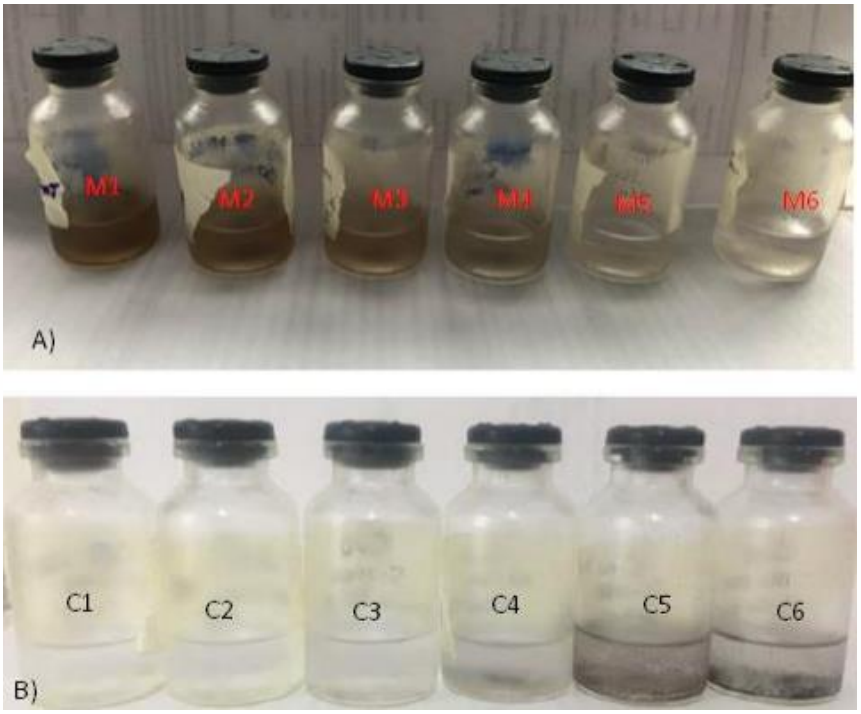

Figure S5. Evidence of color change in suspended Nps from MnO_2 (M1: 80mJ, 10min; M2: 80mJ, 5 min; M3: 50mJ, 10 min; M4: 50mJ, 5 min; M5; 25mJ, 10 min; M6: 25mJ, 5 min) after reduction to Mn^{3+} for electrochromic analyses.

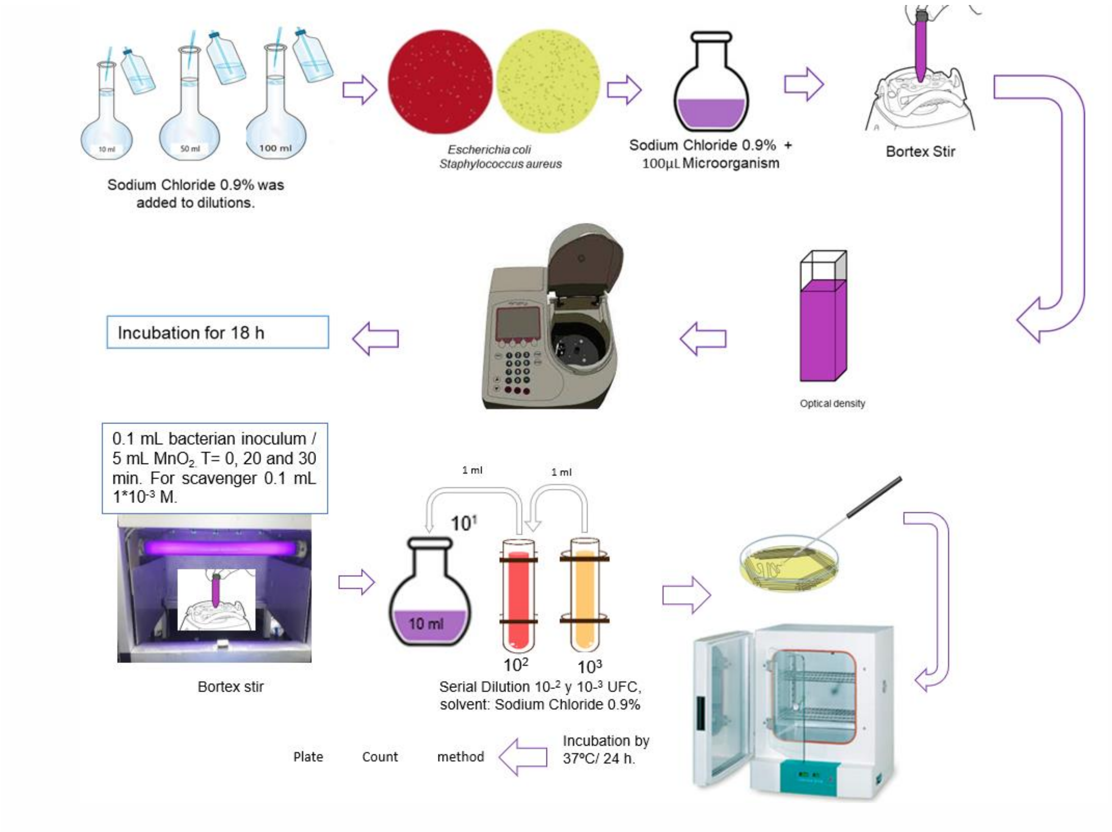

Figure S6. Flow chart of the preparation of the material MnO_2 sterilization mechanism

Table S1. Ventages in top down and bottom-up methods for synthesis of nanoparticles

**Top down methods**			**Bottom-up methods**		
		**Ventages**			**Ventages**
Mechanical milling	Ball milling Mechanochemical method	Enabling chemical reactions in the absence of solvents, therefore resulting in a cleaner synthetic procedure. Obtaining products that	Solid state methods	Physical vapor deposition Chemical vapor deposition	Particle properties of nanostructures such as surface morphology and crystal structure can be controlled.<br><br>Chemical vapor deposition method of coating exhibits the high film durability.

		are difficult or impossible to obtain in the presence of bulk solvents, and expanding the scope of chemical reactions to slightly soluble or inert starting materials.			This method is easy to scale-up.<br><br>Produces nanoparticles of controlled surface morphology.
2	Laser ablation	It is easy to obtain multi-component film that is of the desired stoichiometric ratio by PLD.<br><br>It has high deposition rate, short test period and low substrate temperature requirements.<br><br>Films prepared by laser ablation are uniform.<br><br>The process is simple and flexible with great development potential and great compatibility		Liquid state synthesis methods: Sol gel methods Chemical reduction Hydrothermal method Solvothermal method	Simple method for the formation of thin metal films.<br><br>Particle size and morphology is possible to control by systematic monitoring of reaction parameters<br><br>Desired size and shape nanoparticle can be prepared.<br><br>Well-crystallized powder can be formed.<br><br>Produce nanocrystal with high crystallinity.

		Process parameters can be arbitrarily adjusted, and there is no limit to the type of targets.<br><br>Multi-target components are flexible, and it is easy to prepare multilayer films and heterojunctions.<br><br>It is easy to clean and can prepare a variety of thin film materials.<br><br>Use UV pulsed laser of high photon capability and high energy density as the energy source for plasma generation, so it is non-polluting and easy to control.			
3	Sputtering	The composition of sputtered material is not altered and remains same		Gas phase methods<br>Spray pyrolysis<br>Laser ablation	Relatively simple method.<br><br>Low cost method.

		as that of the target material. Method of choice for refractory metals and intermetallic compounds than other methods like evaporation and laser ablation.<br><br>Economical method as the sputtering equipment is less expensive than electron-beam lithography systems.<br><br>Less impurities are generated than those created by chemical methods.<br><br>Alloy nanoparticles can be produced with easier control on composition than other chemical reduction methods.		Flame pyrolysis	The particle size can be controlled and reproducible.

		This method is a versatile technique to synthesize ionic nanoparticles with spacious sizes and compositions that are not obtainable in solution.<br><br>Slow deposition of heavier ions or mass-selected ions gives unparalleled control of different parameters such as size, composition and charges of ions deposited onto surfaces.			

Table S2. Photocatalytic process for bacteria inactivation using NPs for t= 20 min (suspension, deposited) and 30 min (deposited) of photocatalytic treatment, Units Log UFC

Method			Control BK	Interaction UV	Visible Photolysis	Adsorption	MnO_2 (UV)	MnO_2 (VIS)
**Exposition T=20 min**	**Suspension Nps**	***E.Coli***	2.82	2.54	2.48	1.78	1.08	1.69
		S.Aureus	2.85	2.60	2.47	1.85	1.67	1.39
	**Deposited NPs**	***E.Coli***	2.82	2.29	2.52	2.16	0.69	1.50
		S.Aureus	2.85	2.53	2.37	2.36	1.38	1.25
**Exposition T=30 min**	**Deposited Nps**	***Method***	**Control BK**	**Interaction UV**		**Adsorption**	**MnO_2/UVA**	

	E.Coli	**2.82**	**0.93**	**0.49**	**0**
	S.Aureus	**2.85**	**1.2**	**0.66**	**0.3**
